# Bovine Spongiform Encephalopathy: An Integrated Review of Prion Mechanisms, Neuroanatomy, and Control

**DOI:** 10.3390/vetsci13040398

**Published:** 2026-04-18

**Authors:** Giovanna Pires Marzola, Rodrigo Paolo Flores Abuna, Lucas de Assis Ribeiro, João Paulo Ruiz Lucio de Lima Parra, Matheus Henrique Hermínio Garcia, Sandra Maria Barbalho, Maria Angélica Miglino

**Affiliations:** 1 Regenerative Medicine Laboratory “Carlos Augusto Camargo de Souza Baptista”, Universidade de Marília (UNIMAR), Marília 17525-902, SP, Brazil; giovannapiresmarzola@gmail.com (G.P.M.); rodri_abuna@hotmail.com (R.P.F.A.); joaoparra@unimar.br (J.P.R.L.d.L.P.); matheushenrigarcia@gmail.com (M.H.H.G.); 2 Laboratory of Animal Anatomy, School of Veterinary Medicine, Universidade Federal de Uberlândia, Uberlândia 38408-100, MG, Brazil; lucas.aribeiro@ufu.br; 3 Postgraduate Program in Animal Health, Production, and Environment, School of Veterinary Medicine, Universidade de Marília (UNIMAR), Marília 17525-902, SP, Brazil; 4 Postgraduate Program in Structural and Functional Interactions in Rehabilitation, School of Medicine, Universidade de Marília (UNIMAR), Marília 17525-902, SP, Brazil; smbarbalho@gmail.com

**Keywords:** prion, bovine spongiform encephalopathy, atypical BSE, neuroanatomy, neuroinvasion

## Abstract

Bovine spongiform encephalopathy (BSE) is a fatal transmissible spongiform encephalopathy caused by the misfolding of the host prion protein (PrP), representing a unique intersection between molecular pathology, neuroanatomy, and public health regulation. Although historically framed as a single feedborne epizootic, BSE is now recognized as a spectrum of strain-defined prion disorders encompassing classical and atypical forms with distinct origins, neuroanatomical trajectories, and surveillance implications. This review integrates advances in prion biology, neurodegenerative mechanisms, and anatomical pathways of neuroinvasion to reframe BSE as a heterogeneous disease entity. We synthesize evidence on PrP^C structure, trafficking, and proteolytic processing to explain how normal cellular physiology enables strain-specific conversion to pathogenic PrP^Sc and subsequent neurotoxicity. Distinct patterns of neuroinvasion and regional vulnerability are discussed for classical versus atypical (H- and L-type) BSE, highlighting differences in lymphoid involvement, brainstem targeting, and cortical or cerebellar tropism. We further examine how these biological differences translate into diagnostic sensitivity, surveillance design, and zoonotic risk assessment. By integrating molecular strain diversity with neuroanatomical connectivity, this review underscores the limitations of obex-centered surveillance for atypical BSE and emphasizes the need for proportionate yet precautionary monitoring strategies. These considerations should be interpreted in light of surveillance-dependent detection biases, which influence the apparent distribution of BSE forms. Ultimately, BSE emerges as a critical model for understanding how protein misfolding disorders bridge cellular mechanisms, animal health, and human public health policy.

## Simple Summary

Bovine spongiform encephalopathy, commonly known as “mad cow disease,” is a fatal brain disorder in cattle caused by an abnormal form of a naturally occurring protein. This disease is important not only for animal health, but also because it has caused illness in humans in the past and led to major changes in food safety regulations. Traditionally, bovine spongiform encephalopathy has been treated as a single disease linked to contaminated feed. However, scientific evidence now shows that it actually includes different forms that arise in different ways and affect the brain in different regions. The aim of this review is to explain, in an integrated and accessible manner, how these abnormal proteins form, how they spread within the body, and why different forms of the disease behave differently. We show that some forms mainly begin in the digestive system and spread to the brain through specific nerve pathways, while others may arise spontaneously in the brain of older animals. These differences influence how the disease is detected and monitored. Understanding this diversity is essential to maintain effective surveillance, protect consumers, and prevent future outbreaks. By clarifying how bovine spongiform encephalopathy works at the biological and anatomical levels, this review helps support safer food systems and informed public health policies.

## 1. Introduction

Transmissible spongiform encephalopathies (TSEs), or prion diseases, are uniformly fatal neurodegenerative disorders that, unlike Alzheimer’s or Parkinson’s disease, are infectious: they can be acquired peripherally and subsequently invade the central nervous system (CNS) [1,2]. In animals, they include scrapie in sheep and goats, bovine spongiform encephalopathy (BSE) in cattle, transmissible mink encephalopathy (TME), and chronic wasting disease (CWD) in cervids; in humans, sporadic and variant Creutzfeldt–Jakob disease (sCJD, vCJD), fatal familial insomnia (FFI), Gerstmann–Sträussler–Scheinker syndrome (GSS), and kuru [3,4]. BSE, first recognized in Europe in 1986, is caused by a misfolded, infectious conformer of the host prion protein (PrP), consistent with the “protein-only” hypothesis first proposed by Griffith and later refined by Prusiner [5,6,7,8,9].

Historically, scrapie was the first natural animal TSE described (19th century), characterized by pruritus (“scraping”), ataxia, behavioral change, and a progressive course to death over 3–6 months [4,10]. Scrapie agents persist in pastures for years after removal of infected flocks, and early post-exposure detection in tonsils, retropharyngeal lymph nodes, and intestine supports oral uptake through lymphoid tissues [11,12]. While naturally occurring inherited prion disease syndromes analogous to human pathogenic PRNP-mutation disorders have not been clearly established in animals, PRNP polymorphisms strongly modulate susceptibility (and often incubation period/phenotype) to natural and experimental scrapie [13]. CWD, first noted in captive mule deer in Colorado in the late 1960s, then affected free-ranging populations; saliva-borne infectivity offered a plausible route for efficient horizontal transmission [14,15].

The first UK BSE cases were reported in 1985; epidemiology later identified ingestion of meat-and-bone meal (MBM) containing infected ruminant tissues as the principal transmission route, with exports of cattle and contaminated feed disseminating BSE widely [7,16]. Clinically, BSE presents with behavioral change, hyperesthesia, anxiety, and gait/postural disturbances that mirror the neuroanatomical loci affected [8,11]. After peaking in 1992 (37,316 cases), the World Organization for Animal Health (WOAH/OMSA) coordinated controls to curb the classical BSE epizootic. Feed bans and restrictions on meat-and-bone meal (MBM) reduced horizontal transmission among cattle, while specified-risk material (SRM) removal lowered both cattle-to-cattle and cattle-to-human exposure. Expansion of targeted surveillance increased the detection of existing cases, improving ascertainment without directly reducing transmission. Even so, the public-health impact remained profound: >280,000 cattle were affected over two decades, and in 1996 a new human prion disease, variant CJD (vCJD), emerged in the UK and was causally linked to BSE exposure. Reports of probable human-to-human transmission via blood transfusion further tightened safeguards. In parallel, atypical BSE forms (H- and L-type) detected worldwide raised new questions about pathogenesis, surveillance sensitivity, and residual risk [16,17,18,19,20,21,22,23].

Here, we synthesize clinicopathological, neuroanatomical, and molecular evidence to frame BSE as both a biological and regulatory paradigm of prion disease. By integrating how PrP^C is synthesized, trafficked, and converted into misfolded disease-associated PrP^Sc with the anatomical routes implicated in prion dissemination from peripheral sites to the bovine brain, we highlight how conformational strain diversity underlies distinct disease phenotypes in classical versus atypical BSE. This integrative perspective not only helps clarify open questions regarding prion neurotoxicity and spread but also informs surveillance strategies, risk assessment, and policy decisions aimed at preventing re-emergence and cross-species transmission. In doing so, BSE emerges as a critical model for understanding how protein misfolding can bridge molecular pathology, animal health, and human public health. However, much of the existing literature implicitly treats BSE as a single-entity disease, emphasizing classical BSE while extrapolating its neuroanatomical and epidemiological features to all forms. This approach risks underestimating atypical BSE, whose sporadic origin, distinct strain properties, and altered neurotropism challenge conventional surveillance paradigms. A central unresolved question therefore remains: *how do strain-specific prion properties intersect with neuroanatomical connectivity to shape disease emergence, diagnostic visibility, and zoonotic risk?*

This review advances a unifying framework in which BSE is understood not as a single disease entity but as a set of strain-dependent neuroanatomical processes. Specifically, we propose that differences in prion strain properties (classical vs. atypical) interact with neuroanatomical connectivity to determine (i) routes of neuroinvasion, (ii) regional PrP^Sc accumulation, and (iii) the diagnostic ‘visibility’ of disease under surveillance systems. This framework links molecular strain diversity to practical outcomes in detection, risk assessment, and policy.

## 2. Prion Biology and Pathogenesis

Prion diseases are unique among neurodegenerative disorders in that pathogenesis arises not from an exogenous pathogen or genetic mutation alone, but from the corruption of a normal host protein. Central to this process is the cellular prion protein (PrP^C), a widely expressed yet tightly regulated membrane glycoprotein whose normal biology paradoxically enables its pathogenic transformation. Understanding prion disease therefore requires more than describing the misfolded isoform PrP^Sc; it demands a detailed examination of the physiological structure, trafficking, and processing of PrP^C that permit conformational conversion, neurotoxicity, and intercellular spread [24].

The PrP^C is a membrane glycoprotein anchored by a GPI portion and bearing two variably occupied N-glycosylation sites [25]. The mature ~210-aa protein has a flexible, disordered N-terminus and a compact C-terminal globular domain composed of three α-helices and a short antiparallel β-sheet [26]. In neurons, PrP^C is predominantly diglycosylated and concentrates in lipid-raft microdomains on the outer leaflet of the plasma membrane [27,28]. After endocytosis, it follows two principal itineraries: recycling to the plasma membrane or to the Golgi via retromer-mediated retrograde transport [29,30], or progression to late endosomes/multivesicular bodies (MVBs) for exosomal release or lysosomal degradation [31,32,33,34].

Two proteolytic processing events are especially relevant [35]:

(a) α-cleavage: an internal cut that liberates an unstructured N-terminal fragment while leaving the C-terminal globular domain membrane-anchored [36,37]. Candidate α-secretases (e.g., plasmin, ADAM family) have mixed support, so the responsible protease remains uncertain [38,39,40,41]. This variability reflects differences in experimental systems, and no single protease has been definitively established as the dominant α-cleaving enzyme in vivo.

Although multiple candidate α-secretases have been proposed, the strength of evidence varies considerably. ADAM family proteases are supported by biochemical and cell-based studies, whereas in vivo genetic evidence remains limited and sometimes inconsistent. As a result, no single enzyme can yet be considered definitively responsible for α-cleavage under physiological conditions, and the relative contribution of different proteases likely depends on cellular context.

(b) Ectodomain shedding: a distal C-terminal cut at the cell surface that releases near-full-length soluble PrP; this reaction is mediated by ADAM10 and preferentially targets diglycosylated PrP^C [35,42,43].

***Functions and neural context.*** PrP^C is pleiotropic, with reported roles in neural development [44], cell adhesion [45], axon guidance/synaptogenesis [46], neuroprotection [47], circadian regulation [48], myelin maintenance [49], ion homeostasis [50], and signaling [51]. However, many of these proposed functions derive from in vitro or model-specific systems, and their relative physiological relevance in vivo remains incompletely resolved. Beyond the nervous system, additional activities have been suggested, including immune regulation and copper binding [52]. PrP^C is expressed on long-term repopulating hematopoietic stem cells (self-renewal) [53] and promotes neural precursor proliferation [44]. Postnatal depletion of PrP^C in neurons does not, in itself, cause neurodegeneration [54] underscoring that prion toxicity reflects gain-of-toxic-function, not simple loss of PrP^C. Conversely, cross-linking PrP with certain anti-PrP antibodies can trigger neuronal apoptosis [55].

***Trafficking.*** PrP^C undergoes fast axonal transport (~1 cm/h) and moves both anterogradely and retrogradely [56,57] with transport enriched during axonal regeneration [58]. Several functions map to soluble fragments produced by regulated proteolysis, for example, soluble PrP supports peripheral myelin via Adgrg6/Gpr126 on Schwann cells and promotes neurite outgrowth [59], positioning PrP^C cleavage as a physiological “switch,” conceptually analogous to APP processing [60].

Collectively, these features position PrP^C as a highly dynamic, spatially regulated membrane protein whose normal physiology is closely linked to its pathogenic potential [61,62]. Its association with lipid rafts, axonal trafficking, and tightly controlled proteolytic processing creates multiple opportunities for aberrant conformational conversion, intracellular rerouting, and intercellular spread [63,64,65,66]. The same cleavage events and trafficking pathways that contribute to neurodevelopment, myelin maintenance, and neuronal resilience may therefore be co-opted by misfolded PrP^Sc to promote toxicity and dissemination. Understanding prion pathogenesis thus requires viewing PrP^C not as a static substrate, but as a biologically active protein whose normal cellular itinerary can influence strain-specific neurotropism, incubation time, and disease phenotype [63,64,65,66].

## 3. Classical and Atypical BSE

The recognition that BSE occurs in both classical and atypical forms marked a major shift in prion biology, reframing BSE from a single feedborne epizootic into a heterogeneous group of prion disorders differentiated by strain-associated molecular and neuropathological features [67,68]. These conditions are unified by PrP misfolding but diverge in epidemiology, molecular signatures, and neuroanatomical distribution. This distinction reshaped understanding of BSE origins, transmission dynamics, and residual risk under contemporary control measures [69,70,71]. Whereas classical BSE emerged as a feedborne epizootic driven by anthropogenic practices, atypical BSE appears to arise sporadically, predominantly in older cattle, and is detected primarily through active surveillance [71,72]. Distinguishing these entities is therefore critical for interpreting case detection, refining diagnostic sampling, and informing proportionate risk-management and control strategies.

***Classical BSE (C-BSE).*** The BSE story begins in the United Kingdom in 1986, when cattle developed a fatal neurodegenerative disease linked to feed practices of the time. The agent spread efficiently through MBM given to ruminants, seeding a large epizootic. The turnaround was policy-driven: MBM bans, systematic removal of SRM, targeted surveillance, and robust animal identification/traceability drove the global decline [20,73]. C-BSE typically affects adult cattle (~2–7 years) and shows ready PrP^Sc detection at the obex (medulla, level of the dorsal motor nucleus of the vagus).

***Atypical BSE (H- and L-types).*** Three decades later, active surveillance revealed very rare, apparently sporadic cases of BSE in older cattle across several countries. Because these atypical cases can arise spontaneously and occur at extremely low frequency, they are excluded from WOAH/OMSA classical BSE risk categorization (i.e., atypical detections do not alter a country’s classical BSE risk status). Western blotting after PK digestion separates H and L types by the mobility of the unglycosylated PrP^res band (higher for H-type, lower for L-type); L-type also shows a reduced diglycosylated fraction. A third laboratory-defined profile, “SW” (short incubation, weight loss), has been reported after experimental passage but not in field cases [74,75,76,77]. In atypicals, rostral (e.g., frontal cortex) and cerebellar involvement may exceed the obex burden, so adding the cerebellum and rostral cortex to the sampling improves atypical case capture [78,79,80].

***Resistance and controls.*** BSE prions resist many physical/chemical inactivation methods (freezing, UV, burial, common disinfectants, moderate heat, proteases). Infectivity can persist after 160 °C dry heat for 24 h; standard disinfectants (ethanol, formaldehyde, iodophores, phenolics) are ineffective at typical use conditions. Control, therefore, prioritizes reducing prion stability where possible and, critically, excluding SRM from feed/food chains [81,82,83]. For animal by-products destined for feed, authorized facilities should use OIE-recommended pressure sterilization (133 °C, ≥20 min, 3 bar saturated steam), which can reduce infectivity in MBM by up to 10^3^-fold if prions are present, risk reduction, not sterilization, and must be paired with SRM removal and feed bans [84].

***Atypical BSE as a disease of aging.*** The prevailing view is that atypical H- and L-type BSE represent spontaneously arising, sporadic prion diseases detected mainly in older adult cattle (typically ≥7–8 years), with longer incubation than classical BSE [85].

**Figure 1:**
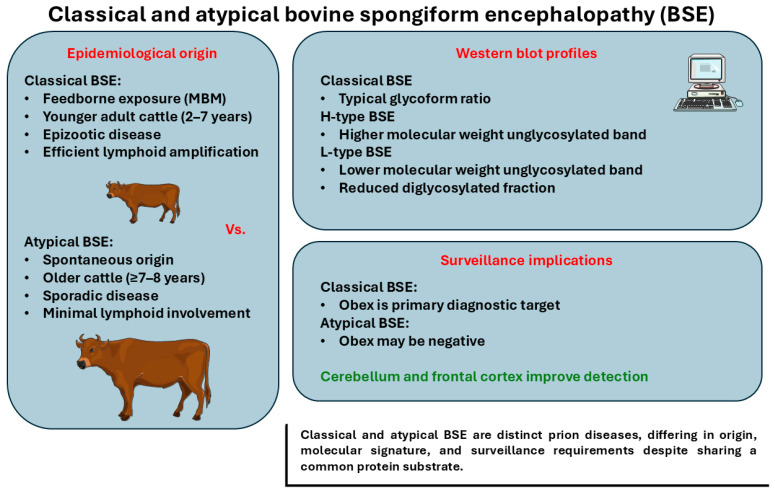
Classical and atypical bovine spongiform encephalopathy: epidemiology and molecular signatures. Classical bovine spongiform encephalopathy (C-BSE) and atypical BSE represent biologically distinct prion diseases. C-BSE is an acquired, feedborne epizootic that typically affects younger adult cattle and is characterized by early involvement of the medullary obex and brainstem autonomic circuitry, consistent with its diagnostic detection at the obex. In contrast, atypical BSE occurs sporadically, predominantly in older cattle, and shows minimal or absent lymphoid involvement. Molecular strain typing by Western blot following proteinase K digestion distinguishes H-type and L-type BSE based on the electrophoretic mobility of the unglycosylated PrP^res band, with L-type also exhibiting a reduced proportion of the diglycosylated glycoform. These molecular and epidemiological differences have direct implications for surveillance design, including consideration of expanded sampling beyond the obex (e.g., cerebellum and more rostral regions) when atypical BSE is suspected. Created with Smart Servier Medical Art (https://smart.servier.com/, accessed on 10 January 2026).

These strain differences establish the biological basis for divergent neuroinvasion routes and neuroanatomical targeting, developed in later sections. Figure 1 summarizes epidemiological, molecular, and surveillance-relevant distinctions between classical BSE and atypical BSE (H- and L-type), including differences in putative origin, age distribution, and PrP^res biochemical profiles. These distinctions are operationally important, as they shape tissue-level PrP^Sc distribution, clinical expression, and the probability of detection under routine surveillance [86,87].

## 4. Mechanisms of Neurodegeneration

**Figure 2:**
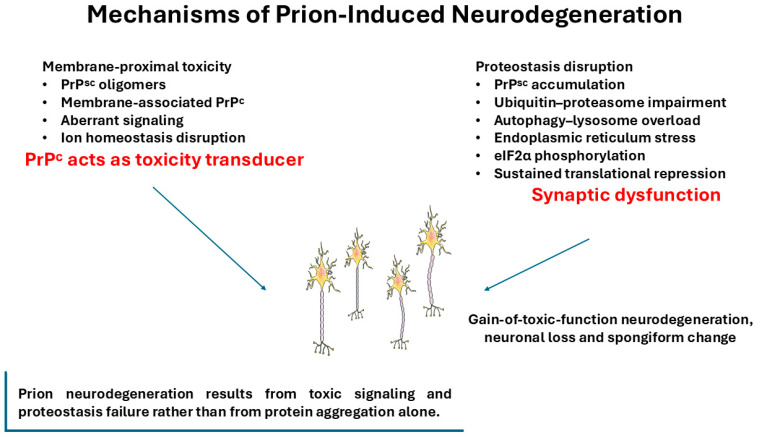
Cellular mechanisms of prion-induced neurodegeneration. Prion neurotoxicity is widely interpreted as a gain-of-toxic-function process involving convergent disruptions of proteostasis and aberrant membrane-proximal signaling. Misfolded, disease-associated PrP^Sc can impair the ubiquitin–proteasome system, overload autophagy–lysosome pathways, and trigger endoplasmic reticulum stress accompanied by sustained eIF2α phosphorylation, resulting in prolonged translational repression, synaptic dysfunction, and progressive neuronal vulnerability. In parallel, small misfolded PrP assemblies may engage PrP^C-dependent membrane signaling networks, promoting abnormal downstream signaling, ion dyshomeostasis, and synaptotoxicity. Together, these interconnected processes contribute to neuronal loss and the spongiform pathology characteristic of prion diseases. Created with Smart Servier Medical Art (https://smart.servier.com/, accessed on 10 January 2026).

Neurodegeneration in prion disease arises not simply from protein aggregation, but from the convergence of conformational templating, intracellular trafficking defects, and toxic signaling events that progressively erode neuronal homeostasis [88,89]. Compared to many other proteinopathies, prion diseases uniquely couple self-propagating misfolded protein states with infectivity, enabling strain-dependent PrP^Sc conformers to impose distinct patterns of cellular stress and neuroanatomical vulnerability [90,91,92]. Dissecting how PrP misfolding drives synaptic dysfunction and neuronal loss is therefore essential for understanding disease phenotypes and the thresholds that separate subclinical infection from overt neurodegeneration [89,93,94]. Figure 2 summarizes the principal cellular mechanisms implicated in prion-induced neurodegeneration, integrating proteostasis disruption, endoplasmic reticulum stress, translational repression, and aberrant membrane-proximal signaling within a gain-of-toxic-function model. It remains unclear whether these processes act sequentially, in parallel, or represent context-dependent manifestations of a common upstream event.

A central event is the templated conversion of PrP^C to a β-sheet-rich PrP^Sc conformer that aggregates [95]. “Steric-zipper” interactions may stabilize β-rich assemblies, and sequence differences within these segments can impose species barriers [96,97]. Conversion is widely proposed to occur along endocytic routes, particularly within multivesicular bodies; however, definitive in vivo localization remains unresolved, and alternative membrane-associated sites have also been suggested [34]. PrP^Sc accumulation is required but not always sufficient for overt neurodegeneration (subclinical states occur) [98]. Conversely, loss of PrP^C alone does not cause degeneration [54]. This dissociation continues to challenge models that directly link aggregate burden to toxicity and suggests that specific conformational species or cellular responses may be more relevant than total PrP^Sc levels.

Two non-exclusive toxicity axes dominate: (i) Proteostasis disruption, early impairment of the ubiquitin–proteasome system, strain on autophagy–lysosome pathways, and persistent ER stress with sustained eIF2α-P–mediated translational repression converge on synaptic failure and neuronal loss; restoring translation is neuroprotective [99,100,101,102]; (ii) Membrane-proximal signaling, oligomeric PrP^Sc associates with neuronal membranes, disturbing receptor function and ion homeostasis; disease accelerates when PrP^C shedding is impaired and when membrane PrP^C acts as a toxicity transducer, paralleling Aβ oligomer signaling via PrP^C–mGluR5–Fyn pathways [103,104,105,106,107,108,109]. Although these frameworks are widely supported, their relative contribution remains debated and may differ across strains and experimental systems.

Among these models, proteostasis disruption is supported by convergent in vivo evidence, including rescue of neurodegeneration through restoration of translational control, suggesting a central role in disease progression. In contrast, membrane-proximal signaling models are strongly supported by mechanistic and in vitro studies but remain less consistently validated across in vivo systems. This disparity indicates that while both pathways likely contribute, their relative importance may differ depending on strain, cell type, and disease stage.

Together, these pathways support a model in which prion neurotoxicity reflects a gain-of-toxic-function process that exploits the normal biology of PrP^C [110,111,112]. Templated conversion can generate neurotoxic PrP assemblies that both overwhelm cellular proteostasis and aberrantly engage membrane-proximal signaling, with conformational variation and cellular context shaping disease tempo and regional vulnerability [94,111,112,113]. Importantly, the frequent dissociation between PrP^Sc burden and neuronal loss suggests that misfolding alone is insufficient: neurodegeneration emerges when adaptive stress responses fail and PrP^C-linked signaling is subverted [112,114,115]. This framework integrates proteostatic collapse, sustained translational repression, and synaptotoxic signaling into a mechanistic continuum, providing a rationale for therapeutic strategies aimed at restoring translational control, trafficking fidelity, and/or PrP^C processing rather than solely reducing aggregate load [111,112,114]. Importantly, the relative contribution of these pathways may vary depending on strain properties and cellular context, underscoring that prion pathogenesis cannot be fully explained by a single mechanistic model. However, the extent to which each pathway contributes to neuronal loss in vivo remains uncertain, and resolving these relationships remains a central challenge in the field. Current evidence therefore favors a multifactorial model in which proteostatic failure represents a core downstream event, while membrane signaling and PrP^C-dependent toxicity act as modulators of neuronal vulnerability rather than sole drivers of degeneration.

## 5. Neuroinvasion and Neuroanatomical Targets: Multiple Entry Modes

**Figure 3:**
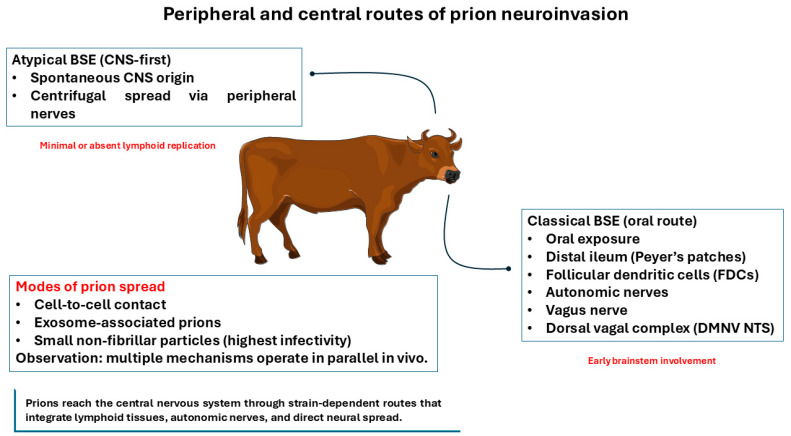
Peripheral and central routes of prion neuroinvasion in BSE. Prion neuroinvasion in BSE can occur through distinct, strain-associated pathways. In classical BSE, oral exposure is followed by uptake within the distal ileum and gut-associated lymphoid tissues (including Peyer’s patches), with subsequent spread via autonomic fibers, most prominently the vagus nerve to the dorsal vagal complex of the medulla, including the nucleus tractus solitarius (NTS) and the dorsal motor nucleus of the vagus (DMV). In atypical BSE, disease is thought to arise sporadically within the CNS, followed by centrifugal dissemination along peripheral nerves with minimal or absent lymphoid involvement. Multiple mechanisms of intercellular prion dissemination may operate in parallel, including direct cell-to-cell contact, extracellular vesicle/exosome-associated transport, and small non-fibrillar prion assemblies that have been associated with high infectivity. Created with Smart Servier Medical Art (https://smart.servier.com/, accessed on 10 January 2026).

Neuroinvasion in prion disease does not follow a single pathway but instead reflects a composite of intercellular transfer mechanisms and anatomically constrained neural routes that together shape incubation time, lesion distribution, and clinical phenotype [116,117]. Prions exploit both cell-associated and cell-free modes of spread, engaging lymphoid tissues and peripheral nerves before reaching defined brainstem and autonomic targets and propagating through connected neuroanatomical networks [118,119]. Understanding how prions move from peripheral sites of exposure to specific CNS targets is therefore essential for linking molecular strain properties to clinical signs, diagnostic yield, and surveillance sensitivity [116,117,118,119]. Figure 3 summarizes the major peripheral and central pathways of prion neuroinvasion in BSE, highlighting strain-associated routes from the digestive tract to the CNS, entry via brainstem/autonomic circuitry, and multiple modes of intercellular prion spread. Here we focus on mechanisms of spread, independent of surveillance or diagnostic considerations.

***Contact-mediated transfer.*** Because prions adhere strongly to surfaces, prion-coated steel wires were used to test whether simple contact could transmit infection. Neuroblastoma cells cultured on those wires became infected, and the same wire then transmitted disease to PrP^C-overexpressing mice after 30 min, with no measurable loss of infectivity [120]. In a complementary model, chronically infected SMB donor cells transmitted infectivity even after aldehyde fixation, indicating direct cell-to-cell transfer [120]. (ii) *Vesicle-bound spread*: exosomes from infected cells can carry prion infectivity (Rov epithelial; Mov neuroglial), and retroviral co-infection boosts release [121,122]; in vivo, exosomes from FDCs in lymphoid tissues may encounter peripheral nerves and seed neuroinvasion [123,124]. (iii) *Cell-free particles:* size-fractionation supports small non-fibrillar particles (~17–27 nm; ~14–28 PrP molecules) as the most infectious species, with large fibrils far less active and ≤5-mers nearly inert [125]. Multiple modes likely operate in parallel in vivo. However, their relative importance under natural conditions remains unclear, as much of the supporting evidence derives from experimental systems. Where PrP^C meets PrP^Sc remains debated; lipid rafts are a leading hypothesis [126]. Fluorescently labeled SAFs visualize uptake in vitro [127], but the most infectious species may be small, non-fibrillar particles [125], so uptake routes may differ by assembly state. Strain diversity may shift uptake, transport, neuroinvasion routes, lesion profiles, incubation time, and lymphoid dependence [4,128,129,130,131]. However, the evidentiary support for these mechanisms is uneven. Direct cell-to-cell transfer and vesicle-associated spread are well demonstrated in controlled experimental systems, whereas definitive in vivo confirmation—particularly under natural exposure conditions—remains limited. Similarly, while small non-fibrillar assemblies show the highest infectivity in biochemical assays, their precise role in physiological transmission and neuroinvasion is still inferred rather than directly observed. Consequently, no single mechanism can yet be considered dominant in vivo, and current models should be interpreted as complementary rather than competing explanations.

***Peripheral to CNS.*** Many TSEs amplify first in lymphoreticular tissues, then invade the CNS via autonomic pathways. In BSE, early deposition and lesions consistently involve the medullary brainstem, nucleus tractus solitarius (NTS), spinal tract/nucleus of V (sp5/spV), vestibular nuclei with secondary involvement of medullary reticular formation, periaqueductal gray, and paraventricular thalamic/hypothalamic regions; cerebellar and cortical changes are typically milder in classical disease [132,133]. This neuroanatomy explains cardinal signs: hyperesthesia/startle (trigeminal/brainstem circuits), postural/balance deficits (vestibular–cerebellar connections), and autonomic disturbances (NTS/DMNV and hypothalamic targets). Oral infection appears to funnel through gut-associated lymphoid tissues and autonomic fibers to the dorsal vagal complex [131,134]. M-cell transcytosis facilitates entry, and enteritis increases susceptibility [135]. Lymphotropic strains rapidly seed Peyer’s patches and nodes [134,136,137], and can reach follicle-bearing organs, enabling urine/milk shedding [138,139,140]. On FDCs and in tingible-body macrophages, complement/CD21/35 facilitates capture [141]; interrupting lymphotoxin signaling dedifferentiates FDCs and can block peripheral replication if done early [142,143,144]. Glycan sialylation may influence complement capture and replication rates and thus interspecies emergence [145,146]. After intracerebral challenge, L-type often shows more rostral (cortical) vacuolation, especially in the frontal cortex, and limited DMNV involvement, consistent with sporadic CNS onset [74,80,147,148,149,150]. H-type frequently yields higher lesion scores in the frontal cortex, thalamus, and hypothalamus than classical disease. In bovine intracerebral studies, lymphoid involvement is minimal/absent for atypicals; peripheral signals, when present, likely reflect late centrifugal spread from CNS along terminal nerves [80,151,152]. Among proposed neuroinvasion routes, the autonomic (particularly vagal) pathway is supported by the most consistent anatomical and lesion-distribution data in natural and experimental BSE, making it the best-substantiated route for classical disease. In contrast, alternative pathways and mechanisms, including hematogenous spread or exclusive lymphoid-independent routes, remain less consistently supported and may be more context- or strain-dependent.

***Sampling targets.*** Highest-yield post-mortem sites include, at the obex, the NTS, sp5/spV, vestibular nuclei, and medullary reticular formation; midbrain periaqueductal gray; paraventricular thalamic/hypothalamic nuclei; and, for atypical surveillance, the cerebellum [80]. Cranial nerve nuclei (V, VII, VIII, IX/X, and II for optic-pathway studies) refine clinicopathologic correlation.

Collectively, these findings support a model in which prion neuroinvasion reflects the intersection of particle/assembly state, cellular context, and neuroanatomical connectivity. Multiple transmission modes may operate in parallel, with strain-associated conformers influencing uptake, lymphoid dependence, and CNS entry routes [113,119,153,154,155,156,157,158,159]. In classical BSE, early involvement of the dorsal vagal complex and related brainstem nuclei is consistent with characteristic clinical features and underpins the diagnostic primacy of the obex, whereas atypical BSE exhibits neuroanatomical patterns compatible with a sporadic CNS origin [67,160]. This mechanistic–anatomical alignment provides a rational basis for tissue sampling strategies and highlights a key operational principle [67,160].

## 6. Neuroanatomy Focus for BSE

Neuroanatomical targeting is a defining feature of BSE and a key determinant of diagnostic sensitivity, zoonotic risk assessment, and surveillance design [161]. Accumulating evidence indicates that classical, H-type, and L-type BSE differ not only in molecular strain properties but also in the regional and cellular distribution of PrP^Sc across the central and, to a more limited extent, peripheral nervous systems [67,68,162,163]. A neuroanatomy-focused perspective is therefore essential for interpreting negative brainstem results, optimizing tissue sampling strategies, and contextualizing experimental transmission data, particularly for atypical forms. Importantly, the strength of evidence underlying these neuroanatomical patterns is not uniform. Brainstem involvement in classical BSE is supported by extensive field, experimental, and diagnostic data, whereas the distribution patterns described for atypical BSE rely more heavily on limited case numbers and experimental inoculation studies. This difference should be considered when extrapolating neuroanatomical findings to surveillance and risk assessment.

Early work on L-type BSE showed that the usual brainstem diagnostic “hot spot” (obex) can be negative in some early cases, while thalamic and olfactory regions carry higher PrP^Sc burdens, a distribution that contrasts with classical BSE [74,164,165] . Transmission studies heightened zoonotic concern: L-type prions tended to transmit more readily and produce faster or more severe disease across models, including human-PrP transgenic mice, cattle, other transgenic mouse lines, and non-human primates, than classical BSE [72,79,165,166]. Given this experimental signal, surveillance strategies for atypicals should remain conservative, even at low prevalence. However, these findings are derived primarily from experimental models, and their direct translation to field risk remains uncertain.

A comparison sampling multiple brain regions, skeletal muscle, and peripheral nerves from classical, H-type, and L-type BSE after intracranial inoculation confirmed distinct brain regional distributions for atypicals; in contrast, peripheral tissues showed no relevant PrP^Sc deposition, mirroring the restriction seen in classical BSE [80,132,167]. Practically, this supports consumer-protection strategies that pair age-threshold rapid testing with brainstem sampling, while acknowledging that atypical surveillance should include cerebellum and rostral cortex to capture L-type cases with reduced obex involvement.

After oral exposure in cattle, the brain, spinal cord, and ileum are the principal sites of accumulation [168]. Muscle can harbor PrP^Sc in classical BSE, whereas adipose tissue tested negative after oral infection or brainstem inoculation [168]. Baseline PrP^C expression is highest in cerebellum, medulla, and spinal cord; intermediate in thymus, intestines, peripheral nerves, heart, spleen; and lower in lung, muscle, kidney, lymph nodes, skin, pancreas, and liver. IHC shows strong labeling in neurons, thymocytes, and lymphocytes with additional signal in the intestinal wall, pancreatic islets, myocardium, alveoli, renal glomeruli, and dermal epithelium [169,170].

***Autonomic pathways and the vagus nerve.*** Converging physiological and pathological data support the notion of autonomic routes for gut-to-brain spread. The vagus nerve appears to be a key conduit during incubation for certain TSEs (including bovine BSE), and heart-rate variability metrics have been proposed as early functional indicators preceding protein-level detection [171]. Although the vagus is not classified as SRM and is difficult to remove completely during processing, PrP^Sc has been detected in the vagus of clinically classical-BSE cattle, relevant for surveillance and risk assessment [171].

***High-sensitivity mapping beyond the brainstem.*** Using tyramide signal amplification (TSA) IHC/IF, PrP^Sc has been detected in the optic nerve (granular labeling in astrocytes, microglia, and periaxonal myelin) and in sympathetic fibers/terminals of the adrenal medulla; PrP^Sc also appears in inferior (nodose) ganglia, sympathetic trunk, vagus, spinal nerves, cauda equina, and again adrenal medulla, reinforcing autonomic and cranial pathways and providing tools to probe H-type pathogenesis [148,151].

***Cell-type tropism across BSE forms.*** Integrating regional and cellular data, L-type preferentially involves CNS neurons early (then PNS), whereas H-type initially targets CNS/PNS glia, with neuronal involvement later; classical BSE follows digestive-tract entry with autonomic neuronal transport to the CNS [163,165].

***Terminology note for diagnostics.*** After PK digestion, the protease-resistant core of PrP^Sc is termed PrP^res (used for Western blot typing), whereas PrP^Sc denotes tissue deposition detected by IHC/IF. We maintain this distinction throughout. The neuroanatomical correlations refine clinical staging and optimize sampling for both classical and atypical BSE.

Together, these data underscore that BSE is best understood as a set of strain-specific neuroanatomical diseases rather than a single uniform entity. Differences in regional vulnerability, autonomic involvement, and cell-type tropism distinguish classical BSE from atypical H- and L-type forms and explain both diagnostic blind spots and apparent differences in experimental transmissibility. Recognition that L-type BSE may evade early obex detection, while preferentially targeting rostral cortical and thalamic regions, reinforces the need for expanded sampling despite its rarity. By integrating high-resolution neuroanatomical mapping with molecular typing, surveillance can remain proportionate yet precautionary, aligning diagnostic practice with the biological realities of prion spread, persistence, and potential zoonotic relevance.

Taken together, strain-dependent neuroanatomical targeting defines not only disease phenotype but also its probability of detection. Classical BSE aligns with obex-centered surveillance, whereas atypical forms—particularly L-type—may remain underdetected due to rostral and cerebellar tropism. Thus, surveillance sensitivity is not solely a function of test performance but of anatomical alignment between sampling strategy and strain biology.

## 7. Clinicopathology and Diagnosis

Clinicopathological features and diagnostic sensitivity in BSE are shaped by strain-associated neuroanatomical targeting and the tempo of disease progression [67]. While classical BSE often presents with recognizable neurologic signs after a prolonged incubation period, atypical forms may be clinically subtle and are most often detected through active surveillance, including fallen-stock monitoring and abattoir testing [67,69]. An integrated understanding of clinical phenotype, neuropathology, and assay limitations is therefore essential for accurate case identification and proportionate surveillance strategies.

Following onset, progression typically takes weeks to months; incubation commonly spans 2–8 years. C-BSE presents with nervousness/hyperesthesia, ataxia/hypermetria (often hind-limb-predominant), reduced milk yield, and weight loss despite preserved appetite [71,172]. Atypical cases are more often detected in fallen stock or at slaughter than via classic neurologic syndromes; described phenotypes include early “apathetic” or “nervous” forms, difficulty rising, and dysmetria [80,150,173,174,175,176].

***Neuropathology and tests.*** No pathognomonic gross lesions. Histology shows bilateral, symmetric spongiform vacuolation in defined brainstem gray-matter nuclei. Diagnosis relies on PK digestion (PrP^C removed; PrP^Sc → PrP^res) followed by WB/ELISA, and IHC (no protease) to localize PrP^Sc in situ; SAFs may be visualized by EM. Strain typing by WB: glycoform/band mobility profiles distinguish types (H higher, L lower mobility of unglycosylated PrP^res; L with reduced diglycosylated fraction; SW lighter than H but experimental) [74,76,79,177].

***Ancillary tests.*** In atypical BSE, ancillary investigations such as cerebrospinal fluid (CSF) analysis and electroencephalography (EEG), even where obtainable, provide nonspecific supportive information at best and lack the sensitivity and specificity required for surveillance or confirmatory diagnosis; consequently, case confirmation continues to rely on post-mortem detection of PrP^Sc in brain tissue [67,178].

***Surveillance.*** Given rostral/cerebellar tropism in some atypical challenges, adding cerebellum (via foramen magnum) and targeted rostral cortex to active sampling can improve detection [80].

In practice, BSE diagnosis relies primarily on post-mortem detection of PrP^Sc rather than clinical presentation alone, particularly for atypical forms that may exhibit variable or attenuated neurologic signs [20,80,84]. The absence of gross lesions and the limited discriminatory value of ancillary clinical tests underscore the central role of biochemical and immunohistochemical assays, coupled with informed tissue selection [178]. As classical BSE incidence declines, maintaining diagnostic sensitivity increasingly depends on adapting surveillance and sampling strategies to the biological features of atypical disease, including consideration of additional sampling beyond the obex (e.g., cerebellum and more rostral regions) to account for strain-associated differences in PrP^Sc distribution [80]. Aligning clinicopathologic interpretation with strain biology therefore remains critical to sustaining confidence in detection systems and minimizing residual risk in contemporary surveillance programs.

## 8. Experimental Transmission, Zoonotic Considerations, and Tissue Distribution

Experimental transmission studies provide a critical lens for evaluating species barriers, strain stability, and potential zoonotic risk in BSE. By comparing transmission efficiency, incubation time, neuropathology, and tissue tropism across animal models and PrP transgenic systems, these approaches help disentangle intrinsic strain properties from host-specific effects and define the biological constraints that govern cross-species propagation [179]. This evidence is particularly relevant for atypical BSE, where experimental transmission has revealed marked differences from classical BSE, including altered strain behavior in heterologous hosts and variable barriers in humanized models [147,167]. Collectively, these findings reinforce the importance of transmission bioassays for translating molecular strain diversity into risk-relevant metrics for surveillance and public-health assessment [147,167].

***L-type.*** In mice, efficient transmission often requires a second passage and shows shorter incubation than classical BSE; with mouse passage, L-type can acquire classical-like biochemical/neuropathologic traits [166,180,181]. In cattle, this convergence has not been observed, even after a second passage [76,80,132]. L-type infects human-PrP transgenic mice (Tg650) with a relatively small species barrier and shows lymphoid tropism in humanized mice; in non-human primates, it produces earlier clinical disease and shorter survival than classical or vCJD, without lymphoid PrP^Sc deposition [72,147,182,183]. Selective muscle atrophy has been reported in some bovine studies [175]. Collectively, these data indicate a higher experimental zoonotic potential for L-type BSE than for classical BSE.

***H-type.*** H-type transmits to WT and bovine/ovine-PrP transgenic mice without adopting a classical phenotype on early passages; with serial passage, classical-like features can emerge in conventional mice and TgBov, and an SW-like phenotype has been observed after specific passage histories [76,103,180,184,185]. H-type does not infect Tg650 humanized mice [147]. In bovine intracerebral challenges, it yields higher vacuolar lesion scores than classical BSE, particularly in the frontal cortex, thalamus, and hypothalamus [186].

***Peripheral distribution.*** In bovine intracerebral studies of atypicals, lymphoid and enteric PrP^Sc are often undetectable; when present peripherally, they likely reflect late centrifugal spread from the CNS along terminal nerves [80,187]. Interpretation is constrained by obex-only surveillance and autolysis in fallen-stock submissions, which may bias detection toward specific disease stages or anatomical distributions, as well as by the age structure and denominators inherent to this surveillance stream [80,148].

Taken together, transmission and tissue-distribution studies indicate that atypical BSE strains, particularly L-type (BASE), are biologically distinct from classical BSE in their interactions with host species and transmission barriers. In multiple experimental systems, L-type BSE shows a comparatively reduced transmission barrier, including efficient propagation in human PrP transgenic mice and transmissibility in non-human primates, supporting heightened *theoretical* zoonotic concern despite its extreme rarity in the field [147,167,188]. In contrast, H-type BSE appears less compatible with humanized models and may display phenotypic plasticity after serial passage in select hosts, consistent with strain adaptation under experimental pressure [76]. Meanwhile, the more limited peripheral detection of PrP^Sc in cattle with BSE, including atypical forms, is consistent with a predominantly CNS-centered disease process and helps explain why case discovery depends heavily on surveillance rather than clinical recognition [67,71]. Interpreted together, these findings support sustained but proportionate surveillance that is grounded in experimental evidence while remaining calibrated to epidemiological reality.

Table 1 shows an outline of the classical and atypical BSE types following the data contained in this article.

## 9. Future Directions

Future research should prioritize high-resolution neuroanatomical mapping of early invasion and tract-to-tract spread in atypical BSE by systematically charting PrP^Sc deposition and lesion topography across the dorsal vagal complex, trigeminal circuitry, vestibulocerebellar pathways, and thalamo-cortical projections using sensitive detection approaches (including tyramide-amplified IHC/IF) and quantitative stereological methods to define first-hit regions and centrifugal versus centripetal propagation patterns [68,189]. Parallel efforts should refine tissue-tropism datasets, particularly cerebellar and cortical burdens in L-type BSE, to update infectivity atlases and, where justified, inform SRM definitions and surveillance sampling algorithms. On the diagnostic front, field-deployable amplification assays such as PMCA and RT-QuIC warrant structured validation for abattoir and on-farm use, coupled with expanded sampling panels that include the obex, cerebellum, and rostral cortex when atypical disease is suspected [190,191,192]. Finally, mechanistic studies should dissect how age-related proteostasis decline, rare PRNP variants, neuron–glia interactions, and synaptic vulnerability converge to drive atypical emergence, providing a framework for interpreting sporadic BSE and guiding preventive strategies [111,114].

Several studies document that atypical BSE cases do not always exhibit maximal PrP^Sc accumulation at the obex and often have substantial amounts of PrP^Sc in more rostral brain regions and the cerebellum. In one comparison of classical vs. atypical BSE, L-type cases showed similar PrP^res signal intensity in the cerebellum compared to the obex, and rostral brainstem regions often had stronger signals than the caudal obex region [193]. Detailed biochemical analyses further show that H- and L-type atypical cases can have relatively even PrP^Sc distribution throughout midbrain, thalamus, and cortical regions, diverging from the more obex-focused distribution seen in classical BSE [165]. Immunohistochemical profiling confirms distinct PrP^Sc patterns across brain areas among BSE types, supporting the value of additional sampling sites for improved detection sensitivity [68].

## 10. Conclusions

Prion diseases illustrate a unique biological paradigm in which misfolded host proteins propagate neurodegeneration across individuals and species. In cattle, classical BSE represents a feedborne epizootic with well-characterized zoonotic consequences, now largely contained through coordinated feed bans, specified risk material (SRM) removal, and comprehensive surveillance. In contrast, atypical BSE arises sporadically in older animals, exhibits distinct neuroanatomical and cellular tropisms, and shows divergent experimental transmissibility, underscoring the need for nuanced sampling strategies and vigilant monitoring despite its rarity [68,172]. While experimental transmission studies, including intracerebral inoculation, transgenic models, and non-human primates, provide critical insights into host susceptibility and strain properties, their relevance to natural exposure scenarios must be interpreted with caution. In particular, intracerebral inoculation bypasses peripheral and mucosal barriers that are central to natural infection processes, and experimental doses are often substantially higher than those encountered under field conditions. Moreover, transgenic and animal models, although valuable for mechanistic understanding, do not fully recapitulate the complexity of species barriers and host–pathogen interactions in natural hosts. Therefore, such studies are best viewed as demonstrating biological plausibility rather than directly reflecting epidemiological risk. Care should be taken when extrapolating these findings to surveillance strategies and assessments of zoonotic potential.

## Figures and Tables

**Table 1:** Comparison of Classical and Atypical Bovine Spongiform Encephalopathy (BSE) Types.

Dimension	Classical (C-Type)	H-Type	L-Type	Integrated Interpretation/Implications
Molecular	Reference PrP^Sc profile	Higher MW PrP^Sc	Lower MW PrP^Sc	Molecular strain differences underpin distinct conformational stability and likely influence neurotropism, transmission behavior, and detectability.
Occurrence	Epidemic, feed-related	Rare, sporadic	Rare, sporadic	Classical BSE reflects external exposure, whereas atypicals likely arise endogenously, implying fundamentally different epidemiological dynamics and control strategies.
Brain regions	Brainstem (obex)	Broader, cortical	Forebrain-predominant	Neuroanatomical targeting diverges significantly, with atypicals showing more rostral involvement, directly affecting diagnostic sensitivity of obex-based sampling.
Clinical/pathology	Typical BSE signs	Atypical signs	Atypical, plaques	Clinical variability reflects underlying neuroanatomical distribution and may reduce clinical recognition of atypical forms.
Diagnosis	Standard	Discriminatory	Discriminatory	Diagnostic performance is optimized for classical BSE; atypicals require expanded sampling (e.g., cerebellum, cortex) and strain typing for accurate detection.
Public health	Zoonotic (vCJD)	No evidence	Uncertain	Divergent experimental transmissibility profiles necessitate a precautionary but proportionate approach, particularly for L-type despite low prevalence.

## Data Availability

No new data were created or analyzed in this study. Data sharing is not applicable to this article.
